# The Function of *Nilaparvata lugens* (Hemiptera: Delphacidae) *E74* and Its Interaction With βFtz-F1

**DOI:** 10.1093/jisesa/ieac041

**Published:** 2022-06-23

**Authors:** Yuwei Zhang, Shiwen Zheng, Yan Li, Xiaojuan Jiang, Han Gao, Xinda Lin

**Affiliations:** College of Life Sciences, China Jiliang University, Hangzhou, 310018, China; College of Life Sciences, China Jiliang University, Hangzhou, 310018, China; College of Life Sciences, China Jiliang University, Hangzhou, 310018, China; College of Life Sciences, China Jiliang University, Hangzhou, 310018, China; College of Life Sciences, China Jiliang University, Hangzhou, 310018, China; College of Life Sciences, China Jiliang University, Hangzhou, 310018, China; College of Biotechnology and Bioengineering, Zhejiang University of Technology, Hangzhou, 310014, China

**Keywords:** brown planthopper, ecdysone, *E74*, *βFtz-F1*

## Abstract

*Drosophila E74* is an early gene located in the polytene chromosome 74EF puff position. *E74* controls the production of late genes, indicating that it plays a crucial role in this cascade model. *Nilaparvata lugens E74* is closely related to *Diaphorina citri*, *Bemisia tabaci,* and *Laodelphax striatellus*. After downregulating *E74*, molting, and nymphal mortality were increased, and ovarian development was delayed. Moreover, the expression of *Vg* was reduced at the transcriptional level, as measured by qRT-PCR, and the content of Vg protein was reduced, as detected by Western blotting. After downregulating *E74*, the expression of hormone-related genes, including *Tai*, *βFtz-F1, Met, Kr-h1*, *UspA*, *UspB*, *E93,* and *Br*, was changed. The expression of *E74* was significantly decreased after downregulating hormone-related genes. When the expression of *E74* and *βFtz-F1* was downregulated together, nymph mortality and molting mortality were higher than those when *E74* or *βFtz-F1* was downregulated alone. Thus, *E74* probably interacts with *βFtz-F1* at the genetic level. In summary, this study showed that *E74* plays a crucial role in the development, metamorphosis and reproduction of *N. lugens*, possibly via the interaction with *βFtz-F1* at the genetic level. This study provides a basis for the development of new target-based pesticides and new methods for the effective control of *N. lugens*.

20-hydroxyecdysone (20E) and juvenile hormone play a decisive role in the development, metamorphosis, and reproduction of insects (Dubrovsky 2005, Mao et al. 2020). 20E is the main steroid hormone in insects, and the Ashburner model for the hormonal control of polytene chromosome puffing indicates that the complex formed by 20E and its receptors controls early puff production (Ashburner et al. 1974, Ashburner 1990). 20E and its nuclear hormone receptor (Usp and ECR) form a heterodimeric complex (Yao et al. 1993), which accurately induces the formation of early puff and inhibits the production of late puff. The protein product produced by early puff induced late puff production at the precise time and inhibited the expression of early puff itself (Ashburner 1990, Fletcher et al. 1995). The 20E-induced early gene *E74* is located on the polytene chromosome 74EF puff (Burtis et al., 1990, Thummel et al. 1990). The E74 homologs from different species have similar structures. E74 belongs to the ETS (E-twenty six) transcription factor superfamily (Sharrocks 2001). *E74* in *Drosophila melanogaster* (*DmE74*) consists of two folded transcription units, *E74A* and *E74B* (Burtis et al. 1990). These two isoforms of proteins share the same C-terminal ETS domain and unique N-terminal domain (Burtis et al. 1990).

The function of *E74* has been extensively studied in many insects. In mosquitoes, yolk formation is tightly controlled by 20E. The *AaE74* isoform, which is homologous to *Drosophila E74B*, is induced after blood feeding, and the peak of *AaE74* transcription coincides with the peak of yolk formation (Sun et al. 2002). In contrast, the *AaE74* isoform homologous to *Drosophila E74A* is activated upon the termination of vitellogenesis. These results suggest that the *AaE74A and AaE74B* subtypes play distinct roles in the regulation of mosquito yolk development (Sun et al. 2002). Metamorphosis is strictly controlled by 20E (Fletcher et al. 1995). In *Drosophila*, when *E74B* was absent, normal pupae could not form and died at the pupal and early pupal stages (Fletcher and Thummel 1995, Fletcher et al. 1995). When *E74A* was deleted, pupae did not die in the pupal stage but died when they molted into an adult (Fletcher and Thummel 1995, Fletcher et al. 1995). After downregulation of *E74* expression, most potato beetles died during the transition from larvae to pupae, and compared with the control, the antennae, legs, and wings of the gene-disrupted potato beetles were shorter (Xu et al. 2018). *E74* also controls programmed cell death of *Drosophila* salivary gland cells and the silkworm (*Bombyx mori*) anterior silk gland (Sekimoto et al. 2007, Wang et al. 2008).

The yolk contains a large amount of yolk protein precursor (YPP), which is secreted into the hemolymph by metabolic tissues such as the fat body, and accumulates in the oocyte (Deitsch et al. 1995). 20E titers increased with vitellogenin production, and when vitellogenin production was complete, 20E titers decreased. Three YPP genes were under the control of 20E: vitellogenin (Vg) (Cho and Raikhel 1992), vitellogenic carboxypeptidase (VCP) (Cho et al. 1991), and vitellogenic cathepsin B (VCB) (Cho et al. 1999). *βFtz-F1* is a decisive factor for the acquisition of competence to 20E (Zhu et al. 2003). In *Aedes aegypti*, downregulation of *βFTZ-F1* attenuated the expression of early response genes, including *E74* and the target *YPP* gene *Vg* (Zhu et al. 2003).

*Nilaparvata lugens* is a notorious migratory rice insect pest in China and Asian countries (Cheng and Zhu 2006, Lin et al. 2018). Persistent infestation of *N. lugens* causes the rice to turn brown and dry, finally leading to ‘hopperburn’ and killing the plant (Cheng and Zhu 2006). In addition, *N. lugens* can transmit the diseases *rice ragged stunt* and *rice grassy stunt* diseases (Cheng and Zhu 2006). The function and mechanism of action of brown planthopper (*N. lugens*) *E74* on *Vitellogenin* (*Vg*) has been reported (Sun et al. 2018); however, this knowledge still not sufficient and the interaction of *E74* with *βFtz-F1* remains unclear. Indeed, the regulation of *Vg* by *E74* in *N. lugens* at both the transcriptional and translational levels also remains unclear. Therefore, this study aimed to downregulate the expression of *E74* by RNAi to study the effect of *E74* on molting, ovarian development, Vg content, and its interaction with *βFtz-F1*.

## Materials and Methods

### Insect and Rice

*Nilaparvata lugens* was originally from Prof. Zengrong Zhu’s laboratory (Insect Research Institute, Zhejiang University, Hangzhou, China) and was later expanded in our laboratory. The culture conditions were 28°C, photoperiod: 14 L:10 D, and relative humidity of 60%. The rice seed used was II You 7954.

### Total RNA Extraction and cDNA Synthesis

Total RNA extraction: nymphs from one to five instars (10–20 for each sample), females and males (10 for each sample) were used. The nymphs were placed into a 1.5-ml EP tube, and 100 µl RNAiso Plus (TaKaRa, Dalian, China) was added; the sample was then ground by a grinder. Then, an appropriate amount of DEPC water was added. The extracted total RNA was measured by a NanoDrop 2000 (Thermo, USA) to evaluate the concentration and integrity.

### First-strand cDNA Synthesis

The Roche Transcriptor First-Strand cDNA Synthesis Kit (Roche Applied Science, Shanghai, China) was used. One microgram of total RNA was used for each cDNA synthesis reaction. The reverse transcription system was performed as described by the manufacturer (Roche).

### Cloning and Sequence Analysis

*E74* primers (forward: 5'GGTGGCCTGTGAAGTAGAGT3', reverse: 5'CGGCTGC AGTTCCATTTTGA3') were synthesized (Sangon Bioengineering (Shanghai) Co., Ltd.). The target gene was amplified by PCR using PrimeSTAR Max DNA Polymerase (Takara, Dalian, China) and purified by a Gel Extraction Kit D2500 (Omega Bio-Tek, GA, USA). The purified fragment was ligated with the pMD-18T vector (Takara) and transformed into Trans5α competent cells (TransGen Biotech, Beijing, China). A single clone was selected and sent to Sangon for sequencing.

MegaAlign 6.0 was used for sequence alignment. The full length of the predicted protein sequence of E74 was aligned with those of other species from NCBI (http://ncbi.nlm.nih.gov).

Molecular Evolutionary Genetics Analysis (MEGA) software 6.0 (MEGA 6.0 software) was used for phylogenetic tree analysis. Maximum likelihood (ML) and neighbor-joining (NJ) were used to construct phylogenetic trees (1,000 replicates). The phylogenetic tree was optimized using iTOL (embl.de).

### dsRNA Synthesis and RNAi

The primers for dsRNA synthesis were designed and are listed in Supp Table S1 (online only). The template was prepared by PCR using PrimeSTAR Max DNA Polymerase (Takara) followed by purification using Gel Extraction Kit D2500 (Omega Bio-Tek). Then, 1,000 µg purified template DNA was added for each reaction. RiboMAX Large-Scale RNA Production Systems SP6 and T7 (Promega, Shanghai, China) were used for dsRNA synthesis. The DNA template was removed by digestion with RQ1 RNase-Free DNase following the transcription reaction. dsRNA synthesis was performed as described by the manufacturer (Promega). The double-stranded RNA was annealed by mixing equal volumes of complementary RNA reactions, incubated at 70°C for 10 min, and then slowly cooled to room temperature for 20 min. Then, 1 µl RNase and 1 µl RQ1 RNase-Free DNase were added to each reaction, which was incubated for 30 min at 37°C to remove any remaining single-stranded RNA and the template DNA. dsRNA was purified by adding 0.1 volumes of 3 M sodium acetate (pH 5.2) and 1 volume of isopropanol or 2.5 volumes of 95% ethanol. After centrifugation and washing with 0.5 ml of cold 70% ethanol, dsRNA was resuspended in nuclease-free water and measured by a NanoDrop 2000 (Thermo).

### RNAi Experiments

Nymphs were anesthetized with carbon dioxide, and a Nikon microscope and Narishige injection system (MN-151, Narishige Scientific Instrument Lab, Tokyo, Japan) were used for injection. Then 0.1µg (0.2µl) of dsRNA was injected into anesthetized *N. lugens*. After 2 h of recovery, the nymphs were transferred and cultured with rice seedlings (Liu et al. 2010, Li et al. 2011).

### qRT-PCR

qRT-PCR primers were designed and are listed in Supp Table S2 (online only). We performed qRT-PCR to compare the stability of different reference genes (*NlRP15* and *NlActin*) after RNAi, by which we selected relative stable reference gene, *NlRP15*, for our qRT-PCR experiments (Supp Fig. S1 [online only]). The kit used for qRT-PCR was Hieff qPCR SYBR Green Master Mix (High Rox Plus) (Yeasen Biotech Co., Ltd., Shanghai, China). A total of 20 μl of the reaction contained 7.2 μl DEPC-treated water, 10 μl SYBR Green dye, 0.4 μl upstream and downstream primers, and 2 μl cDNA template. The reaction program was as follows: 40 cycles of predenaturation at 94°C for 3 min, denaturation at 94°C for 15 s, annealing and extension at 58°C for 40 s. The data used in the detection were all analyzed by the 2^-ΔΔCt^ method (Livak and Schmittgen 2001). Three biological replicates were performed.

### Ovary Dissection and Ovarian Grading

The *N. lugens* nymphs were collected for injection, and were cultured with rice seedlings after injection. Newly eclosed females were paired with three wild-type males, and were dissected and graded 3 d later. The dissection was performed under a stereomicroscope (Nikon, Japan) in PBS. The grading of ovaries was based on a previous publication (Lin et al. 2015).

### Western Blotting

The *N. lugens* nymphs injected with dsRNA were dissected and sampled 3 d after emergence, ground on ice and *centrifuged* for 10 min at 4°C to collect the supernatant. The prepared protein samples were loaded onto an SDS-PAGE protein gel, which was run at 80 V for 30 min and 130 V for 80 min. Then, the protein samples were electrotransfered on ice: constant current 380 mA, transferred to PVDF membranes (ThermoFisher Scientific, Shanghai, China), blocked with 30 ml of skim milk, washed with 1× PBST, and added to diluted Vg antibody (rabbit anti-Vg antibody was a gift from Zhou Qiang of Sun Yat-Sen University, 1:10,000 dilution) and ATCB antibody (Sangon, 1:1,000 dilution) for incubation; after washing, diluted secondary antibody (goat anti-rabbit or horse anti-mouse IgG-HRP, Cell Signaling Technology, Shanghai, China, 1:5,000 dilution) was added, and the samples were washed with 1X PBST (0.1% Tween 20 in PBS). The membrane was placed in the luminescent solution and exposed, and then Image J software was used to calculate the area of the two experimental groups. Three biological replicates were used.

### Imaging and Statistical Analyses

All images were processed with Adobe Photoshop CS5. SPSS 20.0 was used for the statistical analyses. Origin 9.0 was used for the preparation of graphs.

## Results

### Cloning and Sequence Analysis

The amino acid sequence of *N. lugens* E74 has an ETS (E-twenty-six) domain. The comparison of predicted whole amino acid sequences showed that *N. lugens* E74 is conserved with the whole amino acid sequence of *Drosophila melanogaster* (74.68%), *Laodelphax striatellus* (85.01%), *Bemisia tabaci* (65.10%), *Halyomorpha halys* (62.37%), and other species (Supp Fig. S2 [online only]). By comparing predicted amino acid sequences of NlE74 (this study) and NlE74A (previously reported), we noticed two amino acids missing in NlE74A and two amino acids are different between them (Sun et al. 2018; Supp Fig. S3 [online only]).

**Fig. 1.**
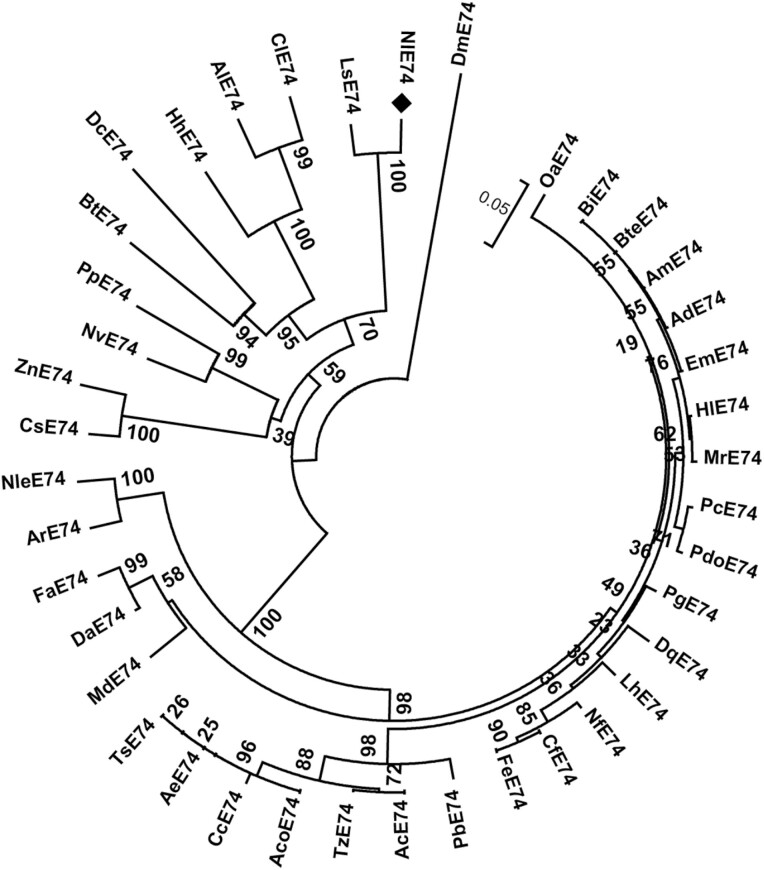
Phylogenetic analysis of E74. NJ method (No. of bootstrap replications = 1,000) was used to construct a phylogenetic tree of NlE74 homologs of different species. *Nilaparvata lugens* (XP_022185398.1); *DmE74*: *Drosophila melanogaster* (NP_730287.1); *AlE74*: *Apolygus lucorum* (KAE9433772.1); *TsE74*: *Trachymyrmex septentrionalis* (KYN41698.1); *LsE74*: *Laodelphax striatellus* (RZF39953.1); *FaE74*: *Fopius arisanus* (XP_011311629.1); *OaE74*: *Orussus abietinus* (XP_012275432.1); *ClE74*: *Cimex lectularius* (XP_014251491.1); *DaE74*: *Diachasma alloeum* (XP_015126943.1); *NleE74*: *Neodiprion lecontei* (XP_015519803.1); *BtE74*: *Bemisia tabaci* (XP_018913898.1); *ArE74*: *Athalia rosae* (XP_020706355.1); *CsE74*: *Cryptotermes secundus* (XP_023704403.1); *HhE74*: *Halyomorpha halys* (XP_024214233.1); *DcE74*: *Diaphorina citri* (XP_026680949.1); *AmF74*: *Apis mellifera* (XP_006558442.1); *AdE74*: *Apis dorsata* (XP_006618775.1); *AeE74*: *Acromyrmex echinatior* (XP_011058740.1); *CfE74*: *Camponotus floridanus* (XP_011265616.1); *PbE74*: *Pogonomyrmex barbatus* (XP_011638318.1); *MrE74*: *Megachile rotundata* (XP_012146095.1); *BteE74*: *Bombus terrestris* (XP_012176122.1); *BiE74*: *Bombus impatiens* (XP_012242920.1); *PdoE74*: *Polistes dominula* (XP_015182724.1); *NvE74*: *Nicrophorus vespilloides* (XP_017770235.1); *AcE74*: *Atta cephalotes* (XP_012057464.1); *AcoE74*: *Atta colombica* (XP_018048966.1); *TzE74*: *Trachymyrmex zeteki* (XP_018317573.1); *PgE74*: *Pseudomyrmex gracilis* (XP_020296097.1); *NfE74*: *Nylanderia fulva* (XP_029166872.1); *FeE74*: *Formica exsecta* (XP_029678077.1); *EmE74*: *Eufriesea mexicana* (OAD61826.1); *LhE74*: *Linepithema humile* (XP_012219554.1); *MdE74*: *Microplitis demolitor* (XP_014295681.1); *DqE74*: *Dinoponera quadriceps* (XP_014481586.1); *PcE74*: *Polistes canadensis* (XP_014602791.1); *HlE74*: *Habropoda laboriosa* (XP_017789379.1); *CcE74*: *Cyphomyrmex costatus* (XP_018394282.1); *ZnE74*: *Zootermopsis nevadensis* (XP_021942781.1); *PpE74*: *Photinus pyralis* (XP_031333759.1).

We built a phylogenetic tree using the NJ method (Fig. 1) and found that *N. lugens* E74 is clustered with *Laodelphax striatellus* E74 and that these two proteins cluster with E74 of *Diaphorina citri*, *Bemisia tabaci*, *Halyomorpha halys*, *Cimex lectularius,* and *Apolygus lucorum* (Fig. 1). It was more distantly related to the amino acid sequences of *Linepithema humile* (54.95%), *Pogonomyrmex barbatus* (48.16%), *Acromyrmex echinatus* (47.72%), and *Trachymyrmex septentrinalis* (54.93%)(Fig. 1). A phylogenetic tree reconstructed utilizing the maximum likelihood (ML) method exhibits a similar topological structure (Supp Fig. S4 [online only]).

### The Spatiotemporal Expression

**Fig. 2.**
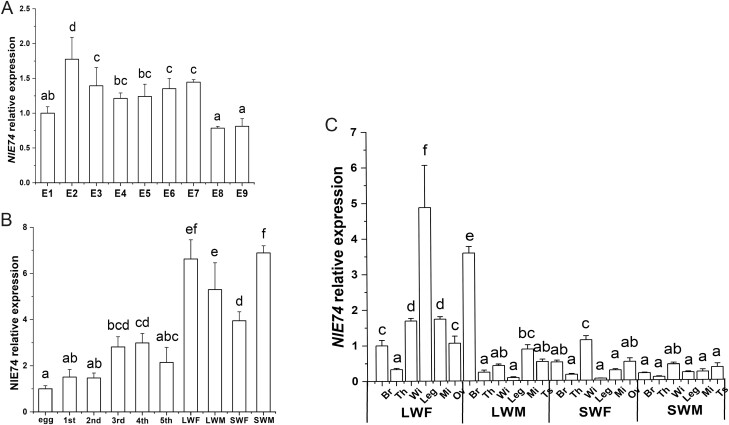
The spatial and temporal expression of *E74*. (A) Expression of *E74* during the embryonic development of *N. lugens*. E1-E9 represent 1–9 d after eggs were laid. (B) Expression of *E74* during development. Egg: egg; 1st: first-instar nymph; 2nd: second-instar nymph; 3rd: third-instar nymph; 4th: fourth-instar nymph; 5th: fifth-instar nymph. (C) Expression of *E74* in different tissues. LWF: long-winged female; LWM: long-winged male; SWF: short-winged female; SWM: short-winged male Br: brain; Th: thorax; Wi: forewing; Leg: leg; Mi: midgut; Ov: ovary; Ts: testis. Three biological replicates were used.

The expression of *E74* was detected in the 1–9 d of the eggs of *N. lugens*, and the results showed that the expression level of *E74* was relatively high in the 2 d after the eggs were laid (Fig. 2A). When the whole developmental stage of *N. lugens* was examined, the expression level of *E74* was higher in the adults than in the eggs and *nymphs (*Fig. 2A and B). We then focused on the expression of *E74* in different tissues of the adults (Fig. 2C). When testing different tissues of adults, we found that the expression of *E74* was relatively high in the brain, forewings, leg, midgut, and ovary of long-winged females. The expression level was higher in the brains of the long-winged males (Fig. 2C).

### Effects of RNAi on the Ovaries

**Fig. 3.**
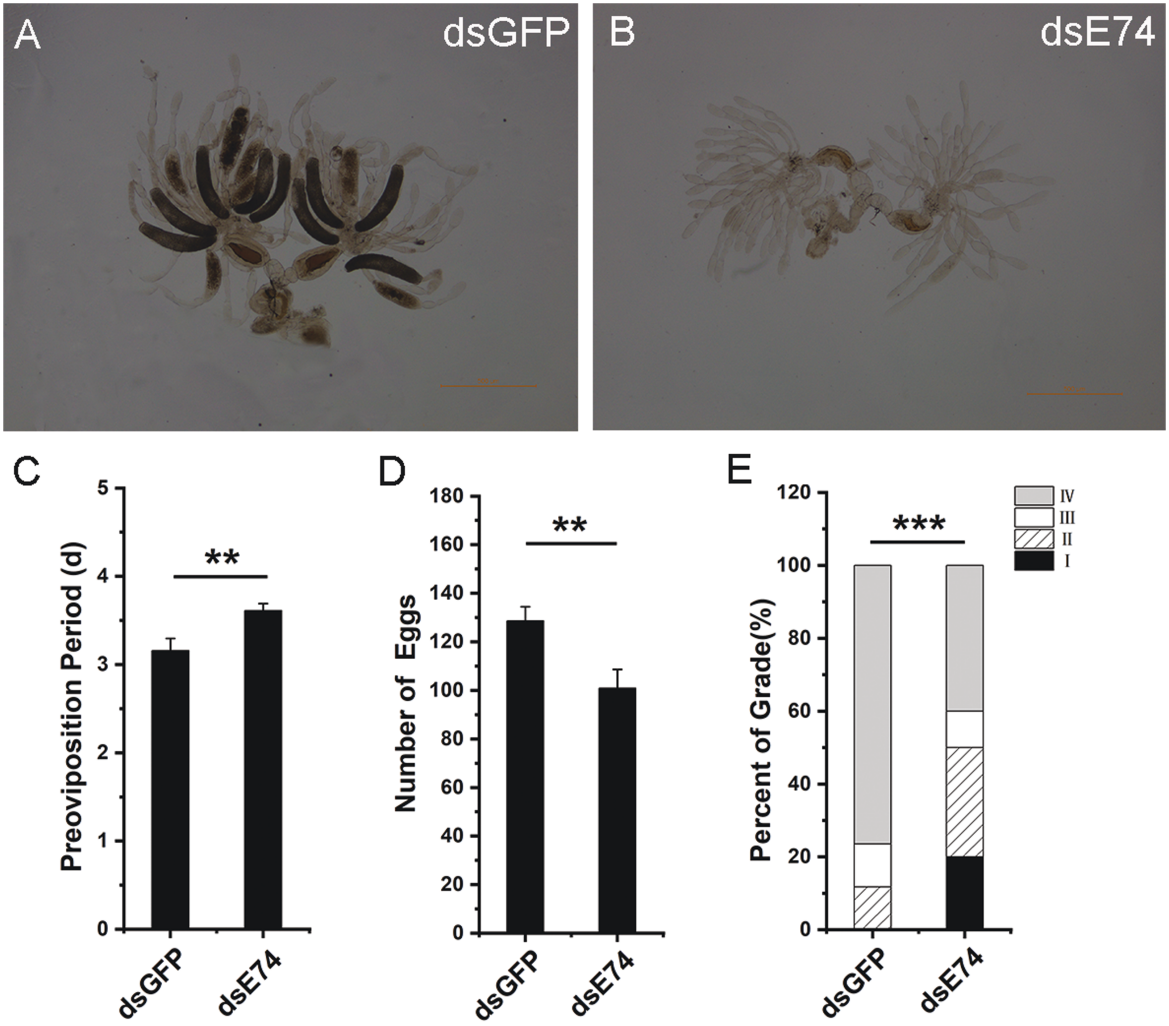
Ovarian development of female *N. lugens*. (A) *N. lugens* ovary injected with dsGFP (control). (B) *Nilaparvata lugens* ovary after downregulating *E74*. (C) The preoviposition period of female *N. lugens* after eclosion. Data analysis was performed using Duncan’s multiple comparisons, and different letters indicate significant differences between the two, *P* < 0.05. (D) The total number of eggs laid by female *N. lugens* after eclosion. Data analysis was performed using Duncan’s multiple comparisons, and different letters indicate significant differences between the two, *P* < 0.05. (E) Ovarian grading of female *N. lugens* after eclosion. I: ovarian grade I; II: ovarian grade II; III: ovarian grade III; IV: ovarian IV; V: ovarian grade V. The chi-square test was used for data analysis, and *** indicates *P* < 0.001. Three biological replicates were used (C–E).

Ovarian development was delayed after downregulating *E74* (Fig. 3A, B, and E). Most of the ovary grades after downregulating *E74* remained grade I, II, and III (Fig. 3A, B, and E). Fifth-instar nymphs were injected with *E74* dsRNA, and after emerging into female adults, they were paired with wild-type males, and the eggs laid on the rice seedlings were counted every day. The preoviposition period was increased significantly after downregulating *E74* (Fig. 3C), which is also consistent with the ovary grading results. Moreover, the number of eggs laid by *N. lugens* after downregulation of *E74* was reduced significantly (Fig. 3D).

### Effect of Downregulating E74 on Vg and Ace Expression

**Fig. 4.**
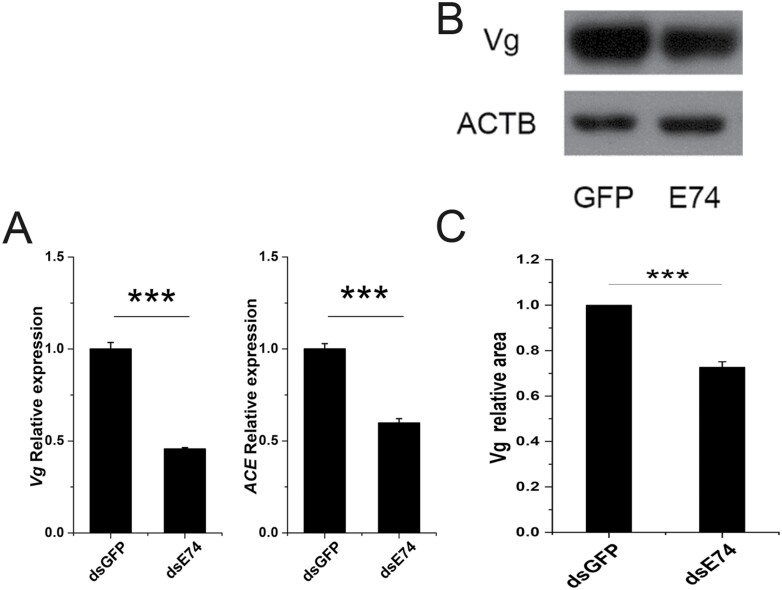
The expression of *Vg* and *Ace* was reduced after downregulating *E74.* (A) The expression of *Vg* and *Ace* measured by qRT-PCR. Data analysis was performed using an independent sample *t* test, * indicates *P* < 0.05. (B) Effect of downregulating *E74* on Vg protein expression. The samples were taken from the abdomen of the female *N. lugens* after downregulating *E74.* The expression of Vg protein was measured by Western blotting; (C) ImageJ was used to quantify the gray value of the bands in B, and the area was calculated. Data analysis was performed using an independent sample *t* test, *** indicates *P* < 0.001. Three biological replicates were used (A, C).

The expression of *Vg* and *angiotensin-converting enzyme* (*Ace*) was measured by qRT-PCR after downregulating *E74*. The transcriptional expression of *Vg* and *Ace* was reduced significantly (Fig. 4A). Moreover, the relative content of Vg protein was detected by Western blotting. After downregulating *E74*, the expression of Vg at the protein level was reduced significantly (Fig. 4B and C). Thus, the effect of downregulating *E74* on *Vg* expression at the transcriptional level was consistent with that at the protein level.

### Expression of E74 After Downregulating Hormone-Related Genes

**Fig. 5.**
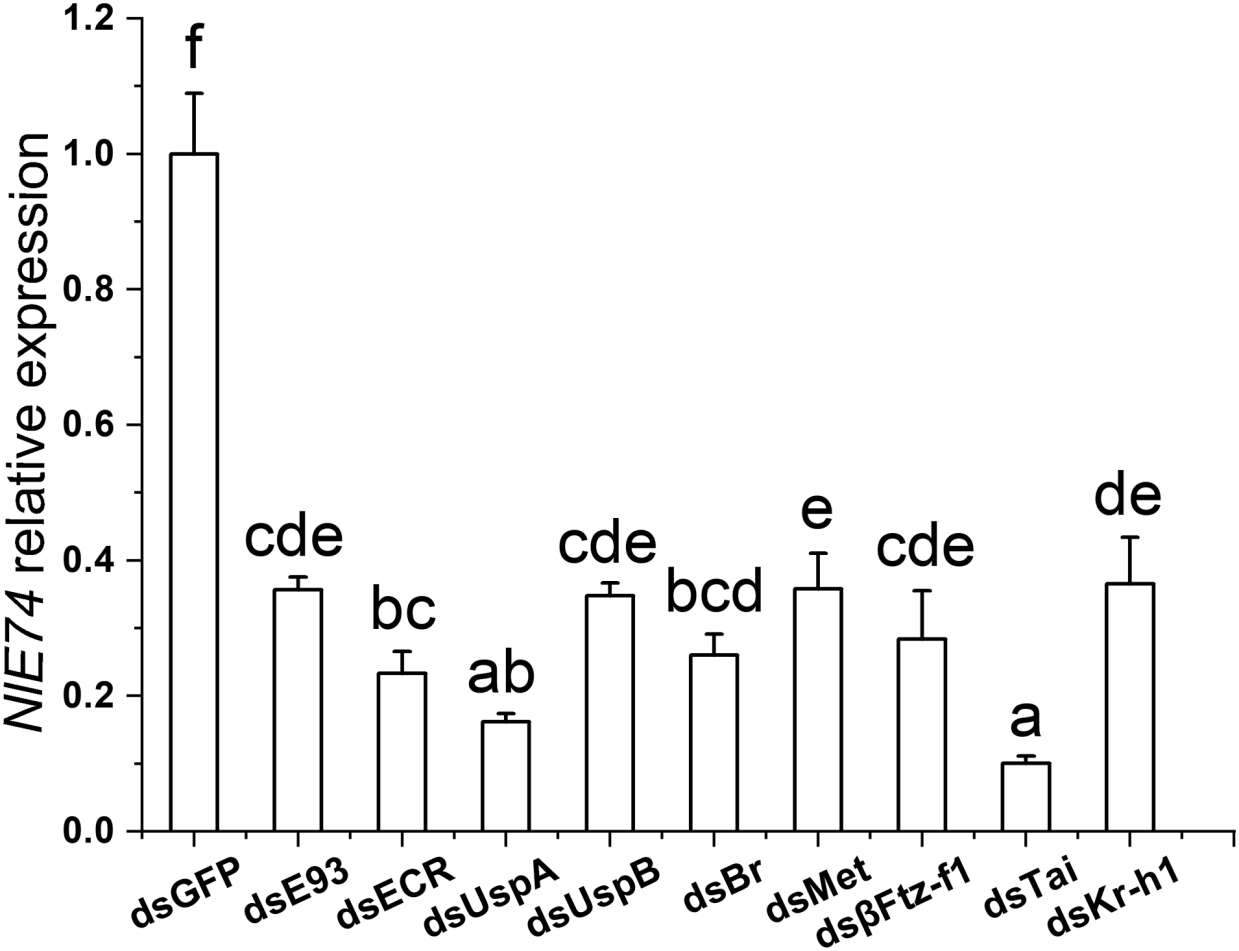
Expression of *E74* after downregulating hormone-related genes. The dsRNAs injected, from left to right: *GFP*, *E93*, *EcR*, *UspA*, *UspB*, *Br*, *Met*, *βFtz-F1*, *Tai*, and *Kr-h1*. The relative expression of *E74* is shown. Duncan’s multiple comparison was used. Different letters indicate significant differences between the two, *P* < 0.05. Three biological replicates were used.

After downregulating of *βFtz-F1*, *Met*, *Tai*, and ds*Kr-h1* (JH signaling pathway) and *E93*, *EcR*, *UspA*, *UspB*, and *Br* (ecdysone signaling pathway), the expression of *E74* in the fifth-instar *N. lugens* nymphs was measured by qRT-PCR. There was a significant change in the expression level: the expression of *E74* was significantly decreased (Fig. 5), suggesting that genes of the JH pathway and the ED pathway had an enhancing effect on the transcription of *E74*.

### Expression of Hormone-Related Genes After Downregulating E74

**Fig. 6.**
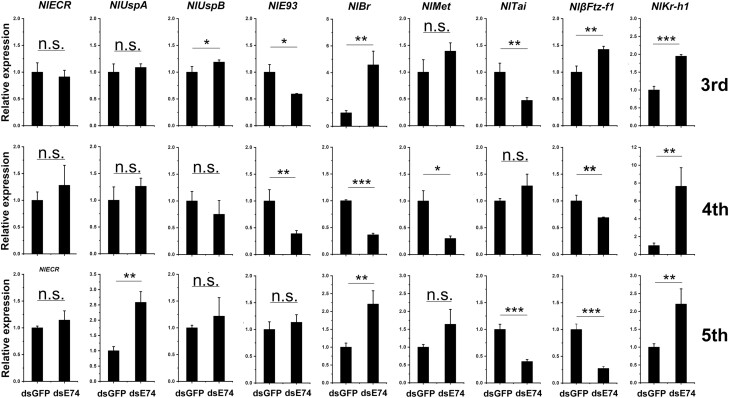
Expression of hormone-related genes after downregulating *E74*. Expression of hormone-related genes in (A) third-instar nymphs injected with *E74* dsRNA; (B) fourth-instar nymphs injected with *E74* dsRNA; (C) fifth-instar nymphs injected with *E74* dsRNA. Data were analyzed by an independent sample *t* test. * indicates *P* < 0.05, ** indicates *P* < 0.001, and *** indicates *P* < 0.001. Three biological replicates were used.

After the third-instar nymphs were injected with *E74* dsRNA, the relative expression levels of *Tai* and *E93* were decreased (Fig. 6), indicating that *E74* had an enhancing effect on the transcription of *Tai* and *E93.* The expression of *Kr-h1*, *UspB*, and *Br* increased (Fig. 6), indicating that *E74* played a role in inhibiting the transcription of these three genes. After downregulating *E74*, the relative expression levels of *EcR*, *UspA*, and *Met* did not change significantly (Fig. 6), indicating that *E74* had no effect on the transcription of these genes.

After fourth-instar nymphs were injected with *E74* dsRNA, the relative expression levels of *Br*, *E93,* and *Met* were decreased (Fig. 6), indicating that *E74* has an enhancing effect on the transcription of *Br*, *E93,* and *Met*. The expression of *Kr-h1* was increased (Fig. 6), indicating that *E74* played a role in inhibiting the transcription of *Kr-h1*. The relative expression of *EcR*, *UspA*, *UspB*, *βFtz-F1,* and *Tai* did not change significantly (Fig. 6), indicating that *E74* had no effect on the transcription of these genes.

After the fifth-instar nymphs were injected with *E74* dsRNA, the relative expression of *βFtz-F1* and *Tai* was decreased, indicating that *E74* has a promoting effect on the transcription of *βFtz-F1* and *Tai*. The expression of *Br* and *Kr-h1* was increased, indicating that *E74* played a role in inhibiting the transcription of *Br* and *Kr-h1*, and the expression of *E93*, *EcR*, *UspB,* and *Met* did not change significantly, indicating that *E74* might not be involved in the transcription of these genes.

### The Effect of RNAi on the Molting

Our qRT-PCR results (Figs. 5 and 6) suggested that *βFtz-F1* is one of the genes that possibly interacts with *E74*. Therefore, we investigated the role of *E74* in molting and the interaction between *E74* and *βFtz-F1*.

**Fig. 7.**
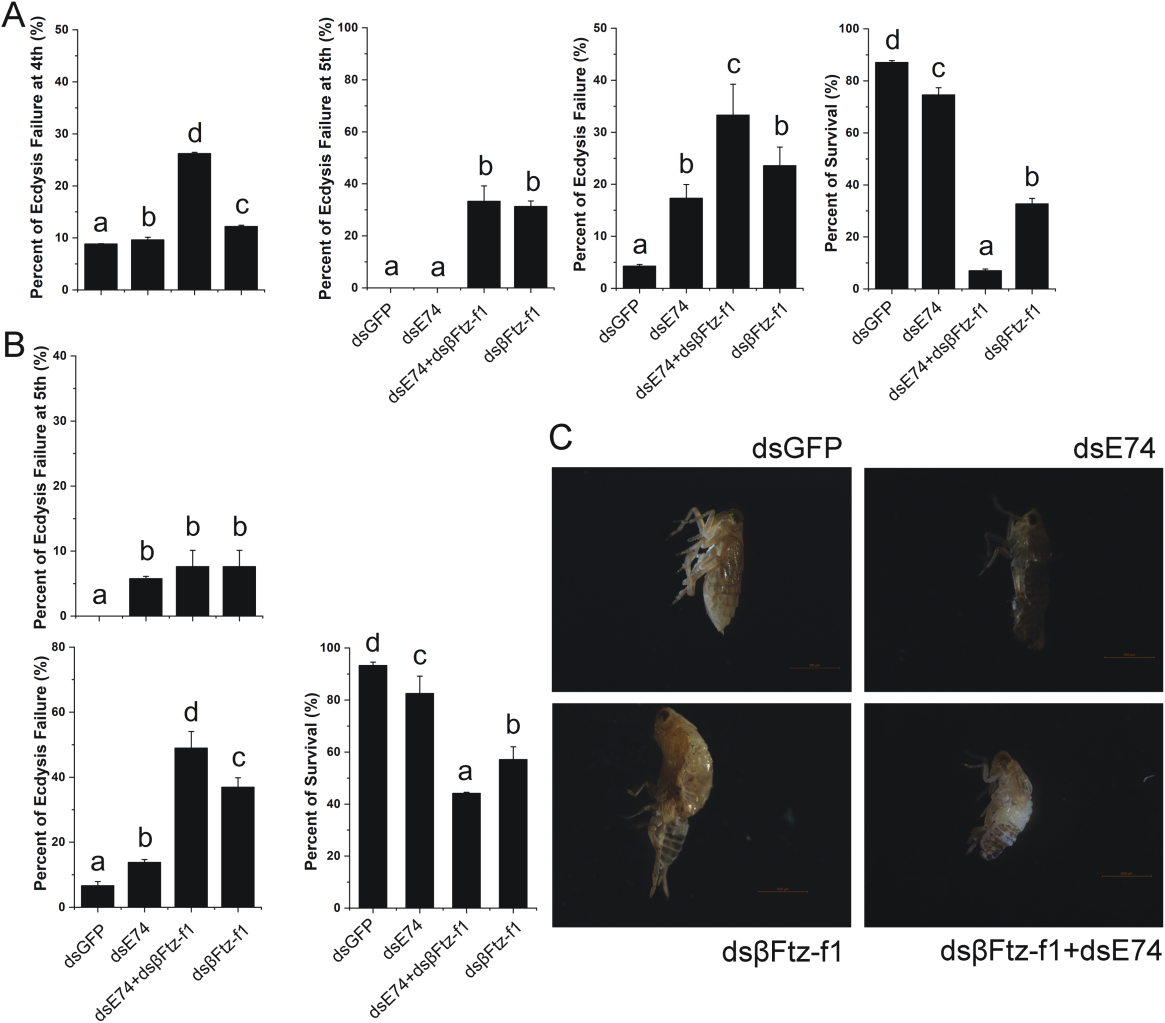
Downregulating *E74* and *βFtz-F1* affect molting. (A) Effect of downregulating *E74* on the molting of the fourth-instar nymphs. (B) Effect of downregulating *E74* on the molting of fifth-instar nymphs. dsRNAs injected: dsE74, dsβFtz-F1, dsE74+dsβFtz-F1, dsGFP. Data analysis was performed using Duncan’s multiple comparisons, and different letters indicate significant differences between the two, *P* < 0.05. Three biological replicates were used (A, B). (C) Compromised molting after RNAi. dsGFP: control; dsE74: fifth-instar nymph molted and died after downregulating *E74*; dsβFtz-F1: fifth-instar nymph molted and died after downregulating *βFtz-F1*; dsE74 + dsβFtz-F1, fifth-instar nymph molted and died after downregulating *E74* and *βFtz-F1* together.

dsRNAs including dsGFP, dsE74, dsβFtz-F1, and dsE74+dsβFtz-F1, were injected into fourth-instar nymphs separately. We found that molting was disrupted after downregulating *E74* and *βFtz-F1* alone or in combination (Fig. 7A). The survival rate of *N. lugens* after downregulating *E74*+*βFtz-F1* was significantly lower than that after downregulating *E74* or *βFtz-F1* alone (Fig. 7A). Moreover, the mortality rate of the fifth-instar nymph after injection of dsE74+dsβFtz-F1 was also significantly lower than that after injection of dsE74 or dsβFtz-F1 alone (Fig. 7B and C). The mortality rate during molting was significantly higher than that of nymphs injected with one dsRNA (Fig. 7B and C).

The expression of *E74* and *βFtz-F1* after downregulating *E74* and *βFtz-F1* in fourth- or fifth-instar nymphs, alone or in combination, was decreased, indicating that RNAi was successful (Supp Fig. S5 [online only]).

## Discussion

When the expression of juvenile hormone and 20E-related genes was downregulated, the expression of *E74* was reduced significantly (Fig. 5), suggesting that both juvenile hormone signaling and ecdysone signaling may affect the transcription of *E74*. However, when the expression of *E74* was downregulated, more juvenile hormone-related genes were affected in transcription than 20E-related genes (Fig. 6), indicating the complex regulatory network of *E74*, which requires further investigation in the future. The cascade model of 20E-regulated polytene chromosome expansion indicates that after 20E forms a complex with its receptors, it can accurately induce the production of early puff. *E74* is located in the polytene chromosome 74EF puff position. *Drosophila E74* is required for metamorphosis and plays a role in a cascade model of 20E-regulated polytene expansion (Fletcher et al. 1995). *βFtz-F1* is an orphan nuclear receptor defining the acquisition of competence to 20E in the mosquito through JH III-mediated posttranscriptional control of *βFTZ-F1* (Zhu et al. 2003). The βFTZ-F1 protein appears after exposure to JH III (Zhu et al. 2003).

Comparing our developmental expression profiles of *NlE74* with a previous report (Sun et al. 2018), we found that the expression profiles were generally similar: for example, *NlE74* was expressed in the first- to fifth-instar nymphs, and the expression level was relatively stable (Fig. 2B). It has high expression in the ovary and midgut of short-winged adults (Fig. 2C). We found that after downregulating *E74* and *βFtz-F1* together, the mortality rate of *N. lugens* during metamorphosis was higher than that of downregulating *E74* and *βFtz-F1* alone (Fig. 7). The expression of *E74* was significantly decreased after downregulating *βFtz-F1* (Fig. 5). Moreover, the expression of *βFtz-F1* was significantly decreased after downregulating *E74* (Fig. 6). These results suggest that *E74* and *βFtz-F1* play critical roles at the transcriptional level in the cascade model of 20E-regulated polytene expansion and likely, metamorphosis. In *Aedes aegypti* females, receptor-bound 20E activates the transcription of early genes, including *E74*, whose protein products are involved in the transcriptional regulation of *YPP* genes such as *Vg* (Roy et al. 2018). The fat body can transcribe a large amount of previtellogenin mRNA. A large amount of yolk precursor protein is produced and transported into the oocyte. We found that the ovaries of *N. lugens* developed slowly after downregulating *E74* (Fig. 3). The number of eggs laid after downregulating *E74* was lower than that of the control (Fig. 3). The expression of *Vg* decreased after downregulating *E74* (Fig. 4A), which is consistent with a previous report (Sun et al. 2018). Moreover, Vg protein levels decreased after downregulating *E74* (Fig. 4B and C). This is consistent with the qRT-PCR results (Fig. 4A). Thus, *N. lugens E74* plays a crucial role in the formation of yolk and reproduction. It is an important link in the cascade model of 20E-regulated polytene chromosome expansion, and likely, in metamorphosis and ovary development. Cis-elements binding Fushi tarazu (Ftz) and E74 were identified within the promoter region of *N. lugens angiotensin converting enzyme* (*NlACE*) (Sun et al., 2018). βFtz-F1 is a co-factor to Ftz (Yussa et al. 2001). We surmise that βFtz-F1 possibly facilitates the binding of Ftz or E74 to the DNA of the promoter region. Furthermore, *E74* and *βFtz-F1* have some common functions (such as regulating molting) (Fig. 7) and regulate the expression of each other (Figs. 5 and 6), and we surmise that they may interact with each other genetically. Future studies, such as on protein–protein interactions, would further help to reveal their interaction.

In summary, we found that *E74* played a vital role in development, metamorphosis and reproduction. The findings also help to further our understanding of the interaction between *E74* and *βFtz-F1*. This study provides better knowledge for the cascade model of 20E-regulated polytene chromosome expansion and provides a basis for the future development of new target-based pesticides and new methods for the effective control of *N. lugens*.

## Supplementary Material

ieac041_suppl_Supplementary_Material
