# Supplementary material for: The Function of Nilaparvata lugens (Hemiptera: Delphacidae) E74 and Its Interaction With βFtz-F1
Source: J Insect Sci. 2022 Jun 23;22(3):15. doi: 10.1093/jisesa/ieac041 (PMC9225820; doi:10.1093/jisesa/ieac041)
Supplement: ieac041_suppl_Supplementary_Material [file ieac041_suppl_supplementary_material.doc]

**Supporting Information**

**Table S1** Primers for dsRNA Synthesis

| **Name** | **Sequence (5’-3’)** |
| --- | --- |
| E93F | TAATACGACTCACTATAGGGAGACCACGCCAGCTTACATGACGAAGA |
| E93R | TAATACGACTCACTATAGGGAGACCACCAGAGTGCAGGATAAATGAC |
| E74F | TAATACGACTCACTATAGGGAGACCACTGGACTGTACACCCCTCACT |
| E74R | TAATACGACTCACTATAGGGAGACCACTGGTCCACTTGATGTACCGC |
| MetF | TAATACGACTCACTATAGGGAGACCACCAACCAGCAGATGAACCTGA |
| MetR | TAATACGACTCACTATAGGGAGACCACGCAAAGCCTCGTACTCTTGG |
| βFtz-f1F | TAATACGACTCACTATAGGGAGACCACCGACCAGATCTCGTTGCTGA |
| βFtz-f1R | TAATACGACTCACTATAGGGAGACCACGCAGCCACAAGTAGAATCCG |
| TaiF | TAATACGACTCACTATAGGGAGACCACTTCATTCATTCAGGCTCGGC |
| TaiR | TAATACGACTCACTATAGGGAGACCACCCACTCACACTACCACCACT |
| Kr-h1F | TAATACGACTCACTATAGGGAGACCACGTGGGGTTCAGTCCTGAGGA |
| Kr-h1R | TAATACGACTCACTATAGGGAGACCACCAGTCGAACACACACCGGAG |
| ECRF | TAATACGACTCACTATAGGGAGACCACCTTCGGTTGGTGGGTCTCTC |
| ECRR | TAATACGACTCACTATAGGGAGACCACGCATTGTCCACCTTCATGCG |
| UspAF | TAATACGACTCACTATAGGGAGACCACTCGGTGGTGCTCTTTTGGTG |
| UspAR | TAATACGACTCACTATAGGGAGACCACAAGTGTGGTGATCTACTGGTCA |
| UspBF | TAATACGACTCACTATAGGGAGACCACTGCTTGTCTTCTTATCATCGCT |
| UspBR | TAATACGACTCACTATAGGGAGACCACGTAAGTGTGGTGATCTACTGG |
| BrF | TAATACGACTCACTATAGGGAGACCACCGTCATCTCGGACAGTGCTA |
| BrR | TAATACGACTCACTATAGGGAGACCACCGAAGTCCCTGAGACAAAGC |
| GFPF | TAATACGACTCACTATAGGGAGATTTGTATAGTTCATCCATGCCATGT |
| GFPR | TAATACGACTCACTATAGGGAGAATGAGTAAAGGAGAAGAACTTTTCA |

**Table S2 Primers for qRT-PCR**

| **Name** | **Sequence (5’)** | **Sequence (3’)** |
| --- | --- | --- |
| *NlE93* | AACAACCTCCCGAAATGCAT | TGCATATGATGGTGGTGGTG |
| *NlE74* | AGCGCAAACTGAAGAAGCA | TGTCGTTGAACCTTCTCTGC |
| *NlMet* | AAAGCCGGTGTCTTTGAAGT | TTTCAGGATTTGGCCGTTCA |
| *NlβFtz-f1* | CCATGAGAACCCGTAATCCG | CACACTCGAGTCCCTTGATG |
| *NlTai* | ATGATCCCAACCACTTCAGC | TTCCACTCACACTACCACCA |
| *NlKr-h1* | TGATGAGGCACACGATGACT | ATGGAAGGCCACATCAAGAG |
| *NlECR* | AAGGCATGTTCCAGCGAAG | GTGTAGGGCTGGTTGTTGG |
| *NlUspA* | CGAGGACTGAGCTTGGAGAA | CTAAAGTTGCCCACCGTGAG |
| *NlUspB* | TGCTTGTCTTCTTATCATCGCT | GTCCCACCGAATTCAACGAC |
| *NlBr* | CCAGGCAAACAACCCAATC | CTACACTGCCCCTCTTCACG |
| *NlActin* | TGGACTTCGAGCAGGAAATGG | ACGTCGCACTTCATGATCGAG |
| *NlRPS11* | CCGATCGTGTGGCGTTGAAGGG | ATGGCCGACATTCTTCCAGGTCC |
| *NlRPS15* | TTCCACGGTTGAAACGTCTGCG | TAAAAATGGCAGACGAAGAAG |


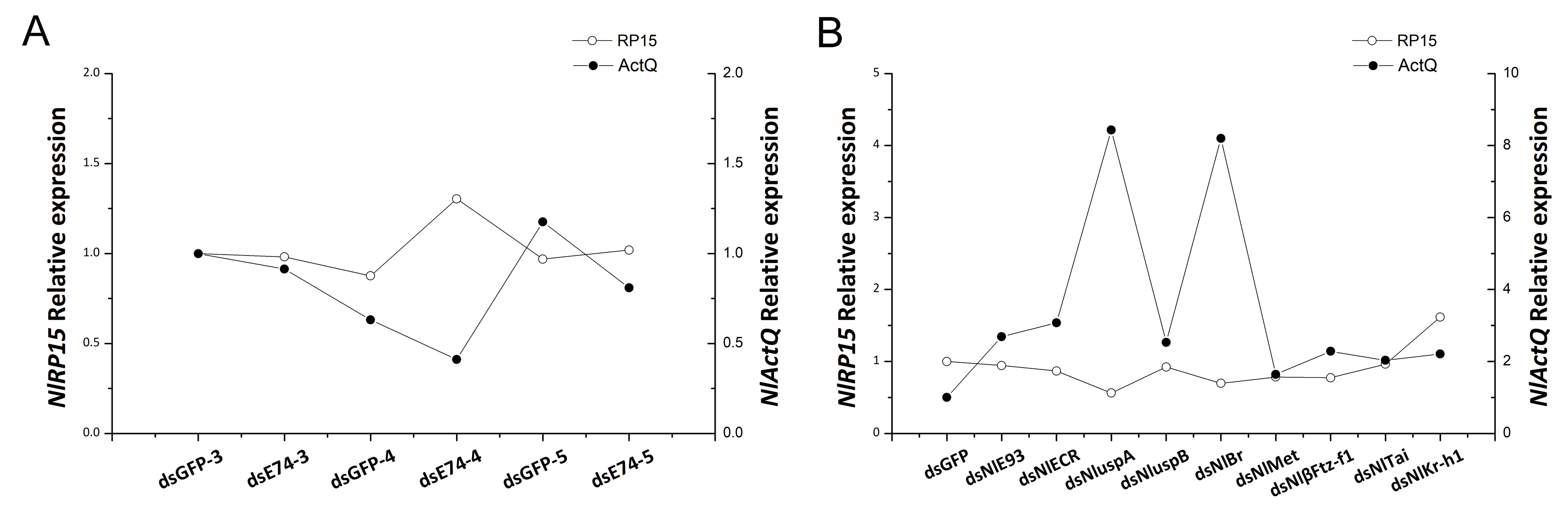


Fig. S1 Stability of the reference genes, *NlRP15* and *NlActin*.

*NlRP11* was used as a reference gene here. A and B are different groups of templates. A, RNAi of *NlE74* at 3^rd^-(dsE74-3), 4^th^- (dsE74-4) and 5^th^-(dsE74-5) instar nymph stage; B, RNAi of different hormone-related genes: *E93*, *EcR*, *UspA*, *UspB*, *Br*, *Met*, *βFtz-F1*, *Tai*, and *Kr-h1*.


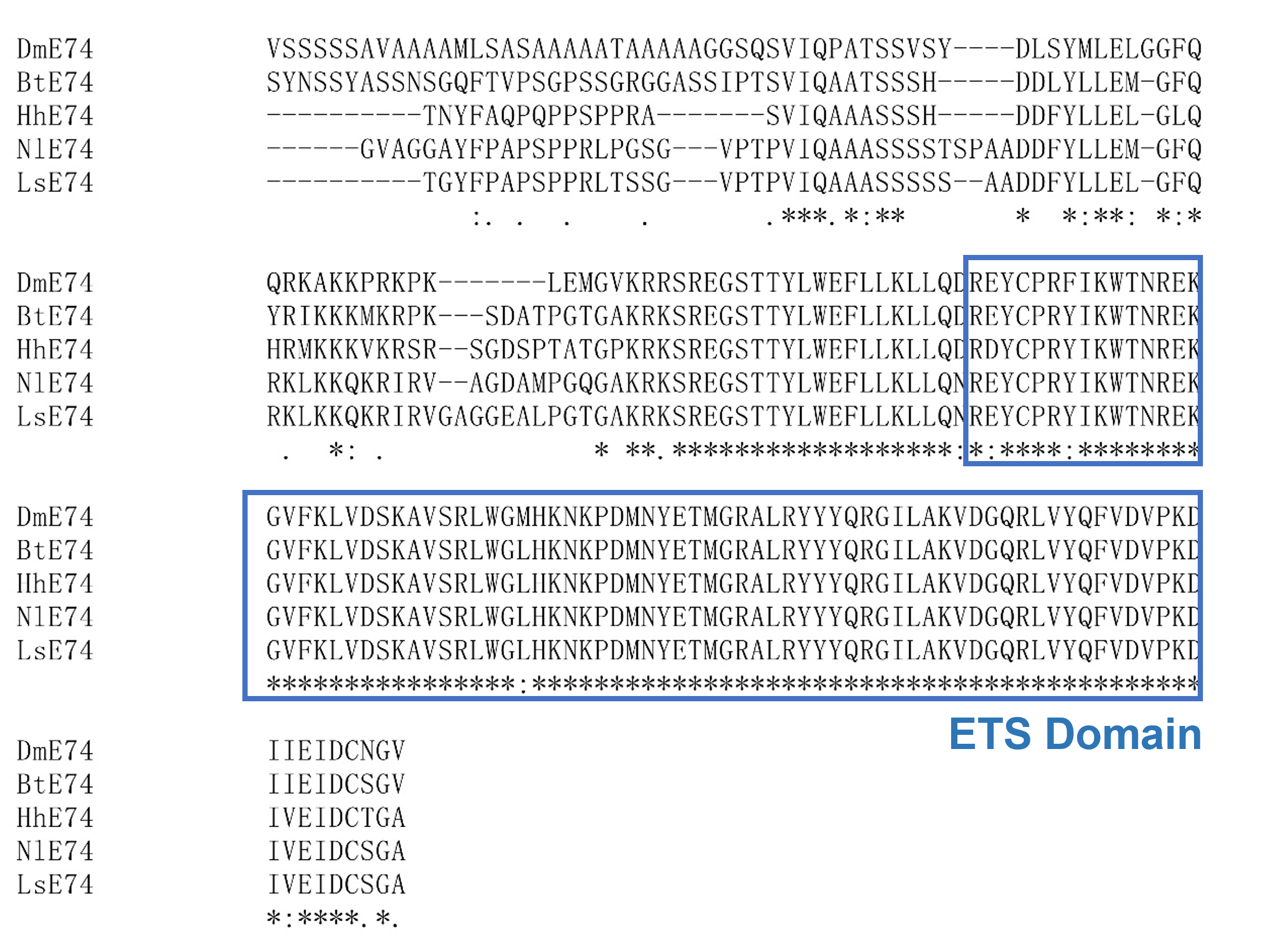


Fig. S2 E74 amino acid sequence analysis. NlE74: *Nilaparvata lugens* (XP_022185398.1); DmE74: *Drosophila melanogaster* (NP_730287.1); LsE74: *Laodelphax striatellus* (RZF39953.1); BtE74: *Bemisia tabaci* (XP_018913898.1); HhE74: *Halyomorpha halys*. Note: The blue box represents the ETS domain.

**
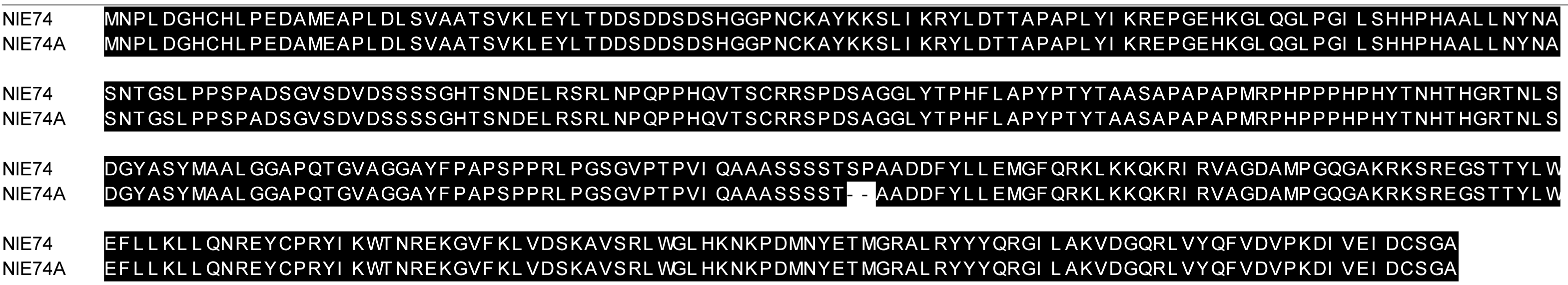
**

Fig. S3 Alignment of NlE74 (this study, XP_022185398.1) and the previous reported NlE74A (KU745459.1).

**
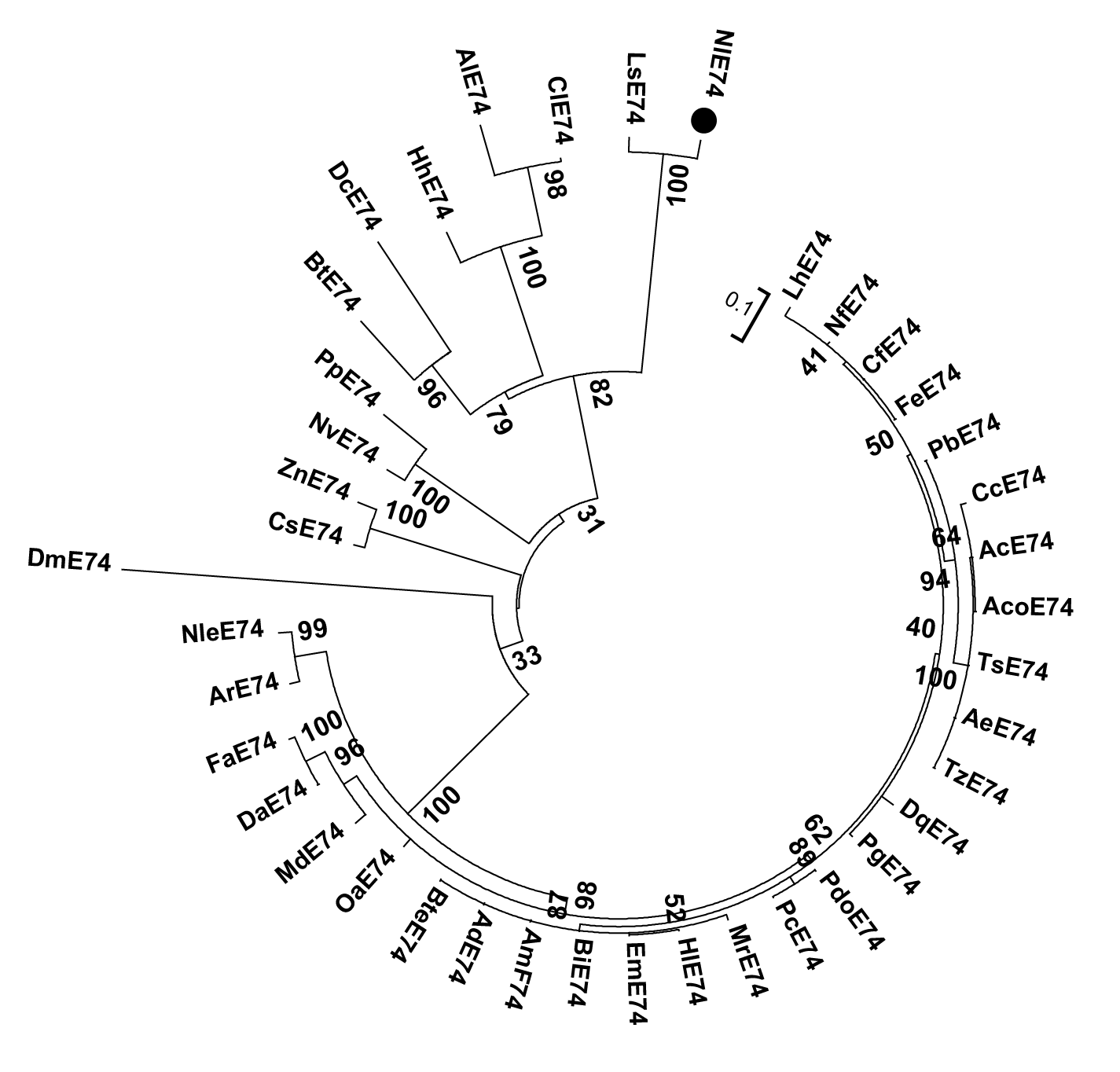
**

Fig. S4 Phylogenetic analysis of E74.

Maximum likelihood method was used to construct a phylogenetic tree of NlE74 homologs of different species. Branch lengths indicate the number of amino acid residue substitutions per site and are drawn to scale.

*Nilaparvata lugens* (XP_022185398.1); *DmE74*: *Drosophila melanogaster* (NP_730287.1); *AlE74*: *Apolygus lucorum* (KAE9433772.1); *TsE74*: *Trachymyrmex septentrionalis* (KYN41698.1); *LsE74*: *Laodelphax striatellus* (RZF39953.1); *FaE74*: *Fopius arisanus* (XP_011311629.1); *OaE74*: *Orussus abietinus* (XP_012275432.1); *ClE74*: *Cimex lectularius* (XP_014251491.1); *DaE74*: *Diachasma alloeum* (XP_015126943.1); *NleE74*: *Neodiprion lecontei* (XP_015519803.1); *BtE74*: *Bemisia tabaci* (XP_018913898.1); *ArE74*: *Athalia rosae* (XP_020706355.1); *CsE74*: *Cryptotermes secundus* (XP_023704403.1); *HhE74*: *Halyomorpha halys* (XP_024214233.1); *DcE74*: *Diaphorina citri* (XP_026680949.1); *AmF74*: *Apis mellifera* (XP_006558442.1); *AdE74*: *Apis dorsata* (XP_006618775.1); *AeE74*: *Acromyrmex echinatior* (XP_011058740.1); *CfE74*: *Camponotus floridanus* (XP_011265616.1); *PbE74*: *Pogonomyrmex barbatus* (XP_011638318.1); *MrE74*: *Megachile rotundata* (XP_012146095.1); *BteE74*: *Bombus terrestris* (XP_012176122.1); *BiE74*: *Bombus impatiens* (XP_012242920.1); *PdoE74*: *Polistes dominula* (XP_015182724.1); *NvE74*: *Nicrophorus vespilloides* (XP_017770235.1); *AcE74*: *Atta cephalotes* (XP_012057464.1); *AcoE74*: *Atta colombica* (XP_018048966.1); *TzE74*: *Trachymyrmex zeteki* (XP_018317573.1); *PgE74*: *Pseudomyrmex gracilis* (XP_020296097.1); *NfE74*: *Nylanderia fulva* (XP_029166872.1); *FeE74*: *Formica exsecta* (XP_029678077.1); *EmE74*: *Eufriesea mexicana* (OAD61826.1); *LhE74*: *Linepithema humile* (XP_012219554.1); *MdE74*: *Microplitis demolitor* (XP_014295681.1); *DqE74*: *Dinoponera quadriceps* (XP_014481586.1); *PcE74*: *Polistes canadensis* (XP_014602791.1); *HlE74*: *Habropoda laboriosa* (XP_017789379.1); *CcE74*: *Cyphomyrmex costatus* (XP_018394282.1); *ZnE74*: *Zootermopsis nevadensis* (XP_021942781.1); *PpE74*: *Photinus pyralis* (XP_031333759.1)

**
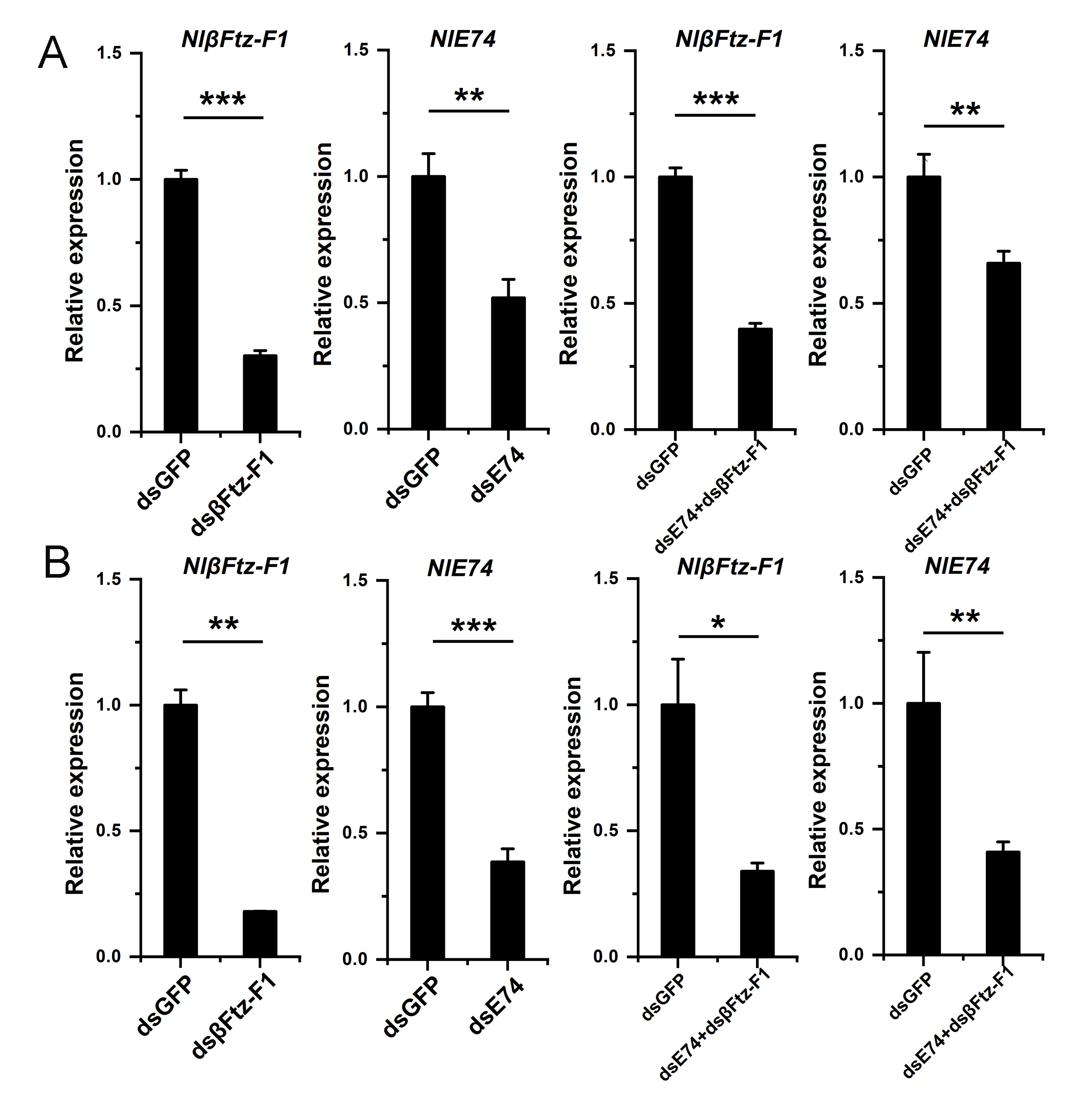
**

Fig. S5 RNAi efficiency of *NlE74* and *NlβFtz-F1*

A, 4^th^-instar nymphs were injected with dsRNA; B, 5^th^-instar nymphs were injected with dsRNA. Student’s *t test* was used. *: P<0.05;**: P<0.01; ***: P<0.001.
